# Genetic Susceptibility to the Environment Moderates the Impact of Childhood Experiences on Psychotic, Depressive, and Anxiety Dimensions

**DOI:** 10.1093/schbul/sbad130

**Published:** 2025-03-04

**Authors:** Neus Barrantes-Vidal, Pilar Torrecilla, Patricia Mas-Bermejo, Sergi Papiol, Marian J Bakermans-Kranenburg, Marinus H van IJzendoorn, Alexia Jolicoeur-Martineau, Thomas R Kwapil, Araceli Rosa

**Affiliations:** Departament de Psicologia Clínica i de la Salut, Universitat Autònoma de Barcelona, Barcelona, Spain; Sant Pere Claver—Fundació Sanitària, Barcelona, Spain; CIBER de Salud Mental, Instituto de Salud Carlos III, Madrid, Spain; Departament de Psicologia Clínica i de la Salut, Universitat Autònoma de Barcelona, Barcelona, Spain; Secció de Zoologia i Antropologia Biològica, Departament de Biologia Evolutiva, Ecologia i Ciències Ambientals, Universitat de Barcelona, Barcelona, Spain; CIBER de Salud Mental, Instituto de Salud Carlos III, Madrid, Spain; Institute of Psychiatric Phenomics and Genomics (IPPG), University Hospital, LMU Munich, Munich, Germany; Department of Psychiatry and Psychotherapy, University Hospital, LMU Munich, Munich, Germany; ISPA, University Institute of Psychological, Social and Life Sciences, Lisbon, Portugal; Department of Psychology, Education and Child Studies, Erasmus University Rotterdam, Rotterdam, the Netherlands; Research Department of Clinical, Education and Health Psychology, Faculty of Brain Sciences, UCL, London, UK; Samsung Advanced Institute of Technology AI Lab, Montreal, Canada; Department of Psychology, University of Illinois at Urbana-Champaign, Champaign, IL, USA; Institut de Biomedicina de la UB (IBUB), Barcelona, Spain; CIBER de Salud Mental, Instituto de Salud Carlos III, Madrid, Spain; Secció de Zoologia i Antropologia Biològica, Departament de Biologia Evolutiva, Ecologia i Ciències Ambientals, Universitat de Barcelona, Barcelona, Spain

**Keywords:** gene–environment interaction, schizotypy, psychosis, childhood adversity, risk factors, resilience

## Abstract

**Background and Hypothesis:**

Gene-by-environment (GxE) studies in psychosis have exclusively focused on negative exposures. However, evidence supports the resilience-enhancing effect of positive factors on psychosis outcome. The Differential Susceptibility (DS) model proposes that common genetic variants may confer not only disproportionate responsiveness to negative environments, but also greater sensitivity to positive, resilience-enhancing conditions. This study is the first to apply the DS model to the expression of subclinical psychosis, employing polygenic risk scores of environmental sensitivity (PRS-ES). PRS-ES were hypothesized to moderate, in a DS manner, associations between childhood adversity and psychosis, affective, and anxiety dimensions in young adults. An exploratory goal examined whether PRS for psychotic-like experiences (PRS-PLE) also showed DS patterns.

**Study Design:**

PRS, schizotypy, PLE, depression, anxiety, and childhood adversity ratings were obtained for 197 nonclinical young adults. LEGIT software for testing competitive-confirmatory GxE models was employed.

**Study Results:**

Results largely supported DS: Individuals high on PRS-ES showed increased subclinical psychosis, depression, and anxiety if they had experienced elevated childhood adversity, and lower symptoms if exposed to low levels of adversity as compared with those with low PRS-ES. Similarly, PRS-PLE moderated the effect of adversity on PLE, positive schizotypy, and depression following the DS model, but only PRS-ES moderation on PLE survived statistical correction.

**Conclusions:**

Our results suggest that genetic DS to the environment is relevant to psychosis, depression, and anxiety. Current debates on reconceptualization of genetic “risk” and resilience may benefit from this insight that support optimistic views on preventative efforts for early detection and intervention.

## Introduction

The psychosis phenotype is expressed across a dynamic continuum where schizophrenia represents the most extreme of a broad distributed behavioral expression of psychosis liability expressed as schizotypy traits and psychotic-like experiences (PLE) in the general population.^1–7^ This extended phenotype ranges from adaptation or minimal dysfunction to frank psychosis and seems to reflect genetic and nongenetic etiological continuity—even if there is discontinuity in terms of impairment and need for care.^8–10^

The presence of PLE not only in the psychosis spectrum but also within traditionally nonpsychotic disorders such as anxiety or depression, supports the notion of psychosis as transdiagnostic.^11,12^ Transdiagnostic research has acknowledged the multidimensionality and evolving nature of mental disorders not only within a single diagnostic spectrum, but across the whole psychopathology spectrum (including nonclinical populations), thus enabling research in risk and protective factors that could be common across diagnostic spectra.^13^ There is phenotypic evidence of this commonality and nonspecificity underlying psychopathology as shown by models that cut across discrete diagnostics such as the Research Domain of Criteria^14^ or the Hierarchical Taxonomy of Psychopathology,^15^ among others.^16–19^

Correspondingly, recent cross-diagnostic genetic studies using Polygenic Risk Scores (PRS) also indicate that a substantial portion of common genetic variants associated with disorder risk are nonspecifically associated with a range of mental disorders, thus representing transdiagnostic risk for mental suffering.^20–23^ Moreover, similar psychosocial factors appear relevant for both psychosis and affective spectra.^24–26^ The limited specificity of genetic and environmental factors along with studies reporting affective dysregulation in the earliest expression of psychosis^27,28^ support the notion of a mental health severity spectrum, with neurodevelopmental impairment driving nonaffective psychosis (eg, schizophrenia), which indexes the most severe endpoint of this continuum.^21^ Gene-by-environment interaction (GxE) studies suggested that “genetic-risk” variants may confer more sensitivity to general psychopathological effects of adverse environmental risk factors,^29–32^ and that the genetic architecture of mental disorders might, in fact, partly reflect the genetics of differential susceptibility (DS) to the environment.

Genetic vulnerability to the environment has traditionally been examined within diathesis-stress frameworks,^33,34^ which propose that individuals carrying genetic-risk variants are more vulnerable to the effects of adversity and more prone to develop psychopathology. Therefore, most GxE research has exclusively focused on negative environmental factors. However, recent studies indicate the impact of positive environmental factors on attenuated psychosis expressions and outcomes. For instance, secure attachment relationships or parental support seem protective against PLE among individuals who had experienced adversity,^35,36^ and social support decreased PLE among discriminated against individuals.^37^ This suggests that GxE models should consider *both* negative and positive environmental factors, as suggested by the DS model.^38–40^ This framework poses that individuals differ in their sensitivity (referred to as susceptibility) to both negative and positive environments, an evolutionarily conserved feature documented also in other species. Thus, individuals traditionally considered to carry greater vulnerability may be better conceptualized as having a greater susceptibility to environmental influences (ie, being more plastic or malleable). It suggests that the same genetic variants and biological or temperamental traits involved in increasing negative effects of risk-promoting experiences also enhance the likelihood of benefiting from positive ones (“for better and for worse”).^39^ Candidate-gene studies have confirmed that genes involved in serotoninergic and dopaminergic systems are likely more open to both supportive and adverse environments.^41,42^ For example, carriers of the short allele (“S”) of the 5-HTTLPR gene have shown to be more affected by negative contexts on antisocial behavior,^43^ neuroticism,^44^ or depressive symptoms^45^ but, crucially, to also benefit more from positive environments and therapeutic interventions as compared with those without S alleles.^46^ Finally, and mirroring the traditional diathesis-stress image, the vantage sensitivity model^47^ poses that certain genetic variants (eg, 5HTTLPR, DRD4) may enhance the likelihood of benefiting from positive exposures without also implying an increase in the susceptibility to negative ones.

DS emerged within developmental psychopathology and has been mostly examined in relation to child psychopathology. This is the first study to examine whether DS applies to the expression of schizotypy and PLE in nonclinically ascertained young adults. The main goal was to test whether Environmental Sensitivity PRS (PRS-ES)^48^ moderated the association of different types of childhood adversity with subclinical expressions of anxiety, depression, and psychosis in a DS manner. It was expected that highly genetically sensitive individuals would show increased subclinical symptoms if they experienced childhood adversity and, at the same time, would report *lower* levels of symptoms if exposed to low or no adversity compared with those genetically less sensitive to the environment. As an exploratory goal, we tested whether a PRS specifically related to PLE in nonclinical samples (PRS-PLE)^49^ also moderated the impact of adversity on transdiagnostic phenotypes following the DS model. Consistent with the notion that sensitivity to the environment is a key transdiagnostic causative factor of mental disorders, and with evidence that PRS-Schizophrenia indexes transdiagnostic risk for mental suffering,^21^ we hypothesized that some variance of the PRS-PLE captures this heightened sensitivity to the environment and would yield a DS pattern. Finally, we hypothesized that the positive dimension of schizotypy (unusual experiences and odd beliefs), but not the negative (flattened affect and disinterest in others and the world), would show a DS pattern for both PRS-ES and PRS-PLE given that the positive dimension of psychosis is more strongly, consistently related to childhood adversity across subclinical and clinical expressions.^50^

## Methods

### Participants

This sample was part of the ongoing Barcelona Longitudinal Investigation of Schizotypy Study (BLISS).^51–53^

At T1 of BLISS, 547 unselected college students were screened with self-report questionnaires. A subsample of 214 participants oversampled for schizotypy scores to ensure enough variance in the construct of interest was selected to conduct in-depth examinations comprising a wide range of interview, questionnaire, and experience sampling methodology measurements (T2). This study uses self-report, interview, and genotype data collected at T2. After genetic quality control, the sample with usable genetic data comprised 197 nonclinical young adults (mean age = 21.90 years, *SD* = 2.4, range 19.3–31.9; 77.2% women).

## Materials and Procedure

### Calculation of PRS

DNA extraction was performed using samples obtained from either saliva or cotton swabs. See details on the genotyping, quality control, and imputation procedures in Supplementary Materials.

PRS were computed by summing the number of risk alleles that individuals carried multiplied by their effect sizes, as reported in a Genome-Wide Association Study (GWAS) of reference. We created a PRS-ES based on Keers et al. GWAS^48^ was conducted with a monozygotic twin sample to capture genetic variants associated with intrapair differences in emotional (internalizing) symptoms. The unique nature of a twin sample genetically identical and sharing basically the same family environment allows us to attribute symptom differences to genetic susceptibility to potentially subtle nonshared environmental factors and, thus, to capture environmental sensitivity as a moderator. PRS-PLE was created in the usual way following Legge et al. GWAS.^49^

We applied the classical Clumping + Thresholding (C+T) method with PLINK v1.9. Independent variants were selected by clumping (*r*^2^ < 0.1 within a 1000 kb window for PRS-ES and *r*^2^ < 0.02 within a 1000 kb window for PRS-PLE) using the 1000 Genomes Project phase 3^54^ as a European linkage disequilibrium (LD) reference panel. 93 494 and 104 891 SNPs for PRS-ES and PRS-PLE, respectively, survived clumping. Consistent with previous evidence using PRS-ES,^55^ we obtained scores with *P*-value thresholds of .001, .01, .05, and .1. For the sake of consistency, and given the lack of previous GxE studies with PRS-PLE, the same thresholds were employed for the secondary exploratory analyses with PRS-PLE. The PRS-ES was computed based on 369 SNPs for *P* < .001; 2819 SNPs for *P* < .01; 11 244 SNPs for *P* < .05; and 19 895 SNPs for *P* < .10. PRS-PLE included 1428 SNPs for *P* < .001; 8815 SNPs for *P* < .01; 26 831 SNPs for *P* < .05; and 40 372 SNPs for *P* < .10.

### Early Adversity

Three complementary measures were used to assess early adversity. The Childhood Trauma Questionnaire (CTQ) Short Form^56^ is a self-report measure capturing subjective reports of sexual abuse, physical abuse, emotional abuse, emotional neglect, and physical neglect. The Interview for Traumatic Events in Childhood (ITEC)^57^ is a semi-structured interview also assessing sexual abuse, physical abuse, emotional abuse, emotional neglect, and physical neglect with follow-up questions assessing age of onset, perpetrator(s), duration, and frequency to calculate composite severity scores for each maltreatment subtype. The semi-structured Childhood Experience of Care and Abuse (CECA)^58^ interview focuses on more objective aspects of childhood experiences. Specifically, parental antipathy, role reversal, parental discord, violence between parents, and bullying subscales were used. Thus, this study combined questionnaire and interview measures of adversity in a complementary manner to increase precision in the assessment of environmental experiences. Specifically, employing in-depth interview-based assessments provides contextualized information, contributes to minimizing bias associated with subjective responding, and allows for probing and clarification, as compared with self-report questionnaires.^57,58^

We computed factor scores based on CTQ, CECA, and ITEC using principal component analysis with an oblique rotation.^59^ Four factors labeled Intrafamilial Adversity, Deprivation, Threat, and Sexual Abuse explained 63% of the total variance. Given the highly skewed distribution of the Sexual Abuse factor, it was excluded from further analyses. The Intrafamilial Adversity factor included loadings of the CECA subscales of Parental Discord, Role Reversal, Violence between Parents, and Antipathy, as well as Emotional Neglect from the ITEC. The Deprivation factor included loadings from the ITEC Physical Neglect and CTQ Physical and Emotional Neglect subscales. Finally, the Threat factor was comprised of loadings from CECA Bullying by Peers, ITEC Emotional and Physical Abuse, and CTQ Emotional and Physical Abuse subscales.^59^

### Phenotypic Measures

#### Psychosis Spectrum.

Schizotypy traits were assessed with the Wisconsin Schizotypy Scales short form (WSS-S)^60^ from which participants were assigned positive and negative schizotypy factor scores.^61^ Positive schizotypy taps magical thinking (α = 0.86) and abnormal perceptual experiences (α = 0.84), whereas negative schizotypy captures social (α = 0.88) and physical (α = 0.80) anhedonia. Subclinical positive PLE were assessed using the frequency score of the Positive subscale (eg, “Do you ever feel as if there is a conspiracy against you?”) of the Community Assessment of Psychic Experiences (CAPE),^62^ which showed a reliability of α = 0.76 in this sample.

#### Affective and Anxiety Spectrums.

Anxiety (α = 0.81) and Depression (α = 0.85) subscales of the Symptom Checklist-90-Revised (SCL-90-R)^63^ were used.

### Statistical Analysis

To fit GxE models and test for DS interactions, we used Version 3.6.3 of the LEGIT package^64^ in R.^65^ In the first exploratory phase, main and interaction effects of the PRS and early adversity on the phenotypic outcome measures were analyzed. In the second phase, interactions yielding significant effects (*P*-values <.05) were examined with the competitive-confirmatory approach^66,67^ to determine the type of GxE interaction. The competitive-confirmatory approach envisions weak and strong versions of each model; thus, we fitted a total of 6 GxE models. The model showing lowest Akaike Information Criterion (AIC) represents the best fit. An interaction is classified as “DS” if (a) reporting lowest AIC and (b) the 95% interval of its estimated crossover point is within observable bounds of the environmental score. In addition, to find a balance between possible fitting ill-conditioned models with near-zero interaction effect and minimize the presence of false positives, LEGIT also examines 4 models excluding the GxE interaction term: (a) Intercept only, (b) gene(s) only, (c) environment(s) only, and (d) gene(s) and environment(s) only. If any of the 4 models without an interaction shows the lowest AIC, the interaction is classified as “no evidence of GxE.” Importantly, this confirmatory approach has been shown to be more powerful than the classic Regions of Significance method^68^ in classifying the type of interaction, especially in smaller samples.^64^

All analyses included the first 2 ancestry-informative principal components from the MDS, Principal Component 1 (PC1) and 2 (PC2), as covariates in the first exploratory phase and were trimmed from the second competitive-confirmatory test phase if they were nonsignificant. We used False Discovery Rate (FDR)^69^ to correct for multiple testing across thresholds of PRS-ES and PRS-PLE for each of the outcome measures.

## Results

Descriptive statistics and Pearson correlations among study variables are presented in Supplementary Materials (Supplementary table 1). One of the criteria to examine the fit of the DS model is that the susceptibility factor (ie, PRS) should not be correlated with the environmental factor (ie, early adversity) or outcomes. PRSs did not correlate with other measures—except for 2 correlations, PRS-ES at threshold *P* < .001 with Intrafamilial Adversity (*r* = 0.18) and PRS-ES threshold *P* < .05 with Threat (*r* = 0.14), both small effect sizes. Main effects of covariates PC1 and PC2 are not reported as they did not show any significant association with outcome variables.

### PRS-ES as a Moderating Susceptibility Factor

As shown in table 1, PRS-ES moderated the association between Intrafamilial Adversity and PLE (thresholds *P* < .001; .05; .10) and between Intrafamilial Adversity and anxiety (thresholds *P* < .05; .10). Subsequent competitive-confirmatory analyses classified the interactions as fitting a DS model, indicating that participants with high PRS-ES showed more PLE and anxiety if they experienced high levels of Intrafamilial Adversity, but also lower PLE and anxiety if not exposed to Intrafamilial Adversity. Only threshold *P* < .10 was best fitted in a diathesis-stress model for anxiety, indicating that individuals with high PRS-ES showed greater anxiety when exposed to high levels of Intrafamilial Adversity. Those with low PRS-ES were relatively unaffected by Intrafamilial Adversity. Significant interactions were also found with Threat for positive schizotypy (threshold *P* < .10) and depression (threshold *P* < .01) consistent with DS.

**Table 1.** Effects of PRS-ES, Childhood Adversity, and Their Interaction on Subclinical Psychosis Spectrum, Anxiety, and Depression

		PRS		Childhood adversity		PRS × Childhood adversity		*R* ^2^	Best GxE model^b^
		Est. (*SE*)	*P*	Est. (*SE*)	*P*	Est. (*SE*)^a^	*P* (*P*_FDR_)		
Psychosis spectrum									
Positive Psychotic-like Experiences (CAPE)									
PRS-ES (*P <* .001)	Intrafamilial adversity	−0.315 (0.336)	.348	0.168 (0.511)	.742	0.857 (0.328)	.010 (.038)	0.088	DS S
	Deprivation	−0.148 (0.333)	.658	1.024 (0.522)	.051	0.27 (0.375)	.457	0.075	
	Threat	−0.245 (0.331)	.460	1.390 (0.532)	.010	0.199 (0.353)	.573	0.112	
PRS-ES (*P <* .01)	Intrafamilial adversity	−0.040 (0.151)	.791	−0.198 (0.818)	.809	0.277 (0.157)	.080	0.068	
	Deprivation	−0.004 (0.151)	.978	0.248 (0.911)	.786	0.237 (0.188)	.208	0.079	
	Threat	−0.059 (0.147)	.688	0.369 (0.813)	.651	0.292 (0.173)	.094	0.121	
PRS-ES (*P <* .05)	Intrafamilial adversity	0.021 (0.072)	.770	−0.454 (0.617)	.462	0.206 (0.069)	.003 (.026)	0.096	DS S
	Deprivation	0.039 (0.072)	.594	0.796 (0.704)	.259	0.069 (0.084)	.417	0.076	
	Threat	0.025 (0.072)	.731	0.607 (0.694)	.383	0.130 (0.082)	.108	0.119	
PRS-ES (*P <* .10)	Intrafamilial adversity	0.029 (0.058)	.619	−0.504 (0.649)	.438	0.167 (0.058)	.004 (.026)	0.094	DS S
	Deprivation	0.032 (0.059)	.593	1.053 (0.726)	.149	0.027 (0.070)	.701	0.073	
	Threat	0.010 (0.058)	.863	1.031 (0.703)	.144	0.059 (0.065)	.369	0.111	
Positive Schizotypy (WSS)									
PRS-ES (*P <* .001)	Intrafamilial adversity	0.034 (0.060)	.572	0.066 (0.092)	.477	0.090 (0.059)	.133	0.059	
	Deprivation	0.055 (0.059)	.355	0.230 (0.093)	.014	−0.005 (0.067)	.944	0.076	
	Threat	0.049 (0.059)	.408	0.159 (0.095)	.095	0.080 (0.063)	.204	0.101	
PRS-ES (*P <* .01)	Intrafamilial adversity	0.013 (0.027)	.635	−0.002 (0.147)	.987	0.037 (0.028)	.193	0.055	
	Deprivation	0.014 (0.027)	.594	0.197 (0.163)	.227	0.006 (0.006)	.853	0.073	
	Threat	0.011 (0.026)	.661	−0.090 (0.144)	.534	0.081 (0.031)	.010 (.115)	0.123	DS S
PRS-ES (*P <* .05)	Intrafamilial adversity	0.017 (0.013)	.186	0.046 (0.112)	.682	0.017 (0.012)	.185	0.065	
	Deprivation	0.018 (0.013)	.171	0.247 (0.125)	.049	−0.004 (0.015)	.812	0.081	
	Threat	0.017 (0.013)	.185	0.035 (0.124)	.778	0.028 (0.014)	.052	0.115	
PRS-ES (*P <* .10)	Intrafamilial adversity	0.015 (0.011)	.152	0.087 (0.118)	.463	0.009 (0.010)	.392	0.060	
	Deprivation	0.014 (0.010)	.172	0.310 (0.129)	.017	−0.009 (0.012)	.444	0.084	
	Threat	0.012 (0.010)	.255	0.089 (0.125)	.477	0.017 (0.012)	.151	0.107	
Negative Schizotypy (WSS)									
PRS-ES (*P <* .001)	Intrafamilial adversity	0.050 (0.074)	.498	0.023 (0.113)	.836	−0.056 (0.072)	.440	0.028	
	Deprivation	0.034 (0.071)	.634	0.198 (0.113)	.081	0.012 (0.081)	.882	0.062	
	Threat	0.012 (0.071)	.868	0.316 (0.115)	.007	−0.055 (0.076)	.470	0.082	
PRS-ES (*P <* .01)	Intrafamilial adversity	−0.025 (0.033)	.451	-0.190 (0.178)	.288	0.035 (0.034)	.303	0.032	
	Deprivation	−0.042 (0.032)	.198	0.500 (0.195)	.011	−0.063 (0.040)	.120	0.078	
	Threat	−0.042 (0.032)	.186	0.420 (0.177)	.019	−0.036 (0.038)	.338	0.092	
PRS-ES (*P <* .05)	Intrafamilial adversity	0.020 (0.016)	.204	−0.068 (0.137)	.621	0.004 (0.015)	.787	0.032	
	Deprivation	0.017 (0.016)	.275	0.279 (0.151)	.066	−0.010 (0.018)	.575	0.068	
	Threat	0.013 (0.016)	.423	0.300 (0.152)	.049	−0.007 (0.018)	.679	0.084	
PRS-ES (*P <* .10)	Intrafamilial adversity	0.017 (0.013)	.183	−0.067 (0.143)	.641	0.003 (0.013)	.785	0.033	
	Deprivation	0.016 (0.013)	.220	0.249 (0.156)	.112	−0.005 (0.015)	.761	0.069	
	Threat	0.013 (0.013)	.319	0.159 (0.152)	.299	0.009 (0.014)	.518	0.086	
Anxiety (SCL-90-R)									
PRS-ES (*P <* .001)	Intrafamilial adversity	0.032 (0.391)	.934	0.785 (0.595)	.188	0.431 (0.381)	.260	0.061	
	Deprivation	0.207 (0.388)	.595	1.276 (0.607)	.037	−0.043 (0.436)	.922	0.052	
	Threat	0.098 (0.381)	.797	1.564 (0.609)	.011	0.184 (0.405)	.650	0.104	
PRS-ES (*P <* .01)	Intrafamilial adversity	−0.014 (0.174)	.935	−0.092 (0.938)	.922	0.294 (0.181)	.105	0.068	
	Deprivation	0.015 (0.176)	.934	0.997 (1.062)	.349	0.054 (0.219)	.805	0.051	
	Threat	−0.038 (0.170)	.822	0.947 (0.939)	.314	0.200 (0.201)	.320	0.108	
PRS-ES (*P <* .05)	Intrafamilial adversity	0.062 (0.083)	.451	−0.357 (0.708)	.614	0.217 (0.079)	.007 (.079)	0.096	DS S
	Deprivation	0.083 (0.084)	.327	1.286 (0.817)	.117	−0.010 (0.098)	.917	0.055	
	Threat	0.052 (0.083)	.533	1.688 (0.803)	.037	0.008 (0.094)	.928	0.105	
PRS-ES (*p<*.10)	Intrafamilial adversity	0.058 (0.068)	.392	0.001 (0.751)	.999	0.134 (0.067)	.047 (.283)	0.078	Diathesis-stress S
	Deprivation	0.058 (0.069)	.399	1.824 (0.841)	.031	−0.066 (0.081)	.417	0.057	
	Threat	0.033 (0.067)	.629	2.145 (0.810)	.009	−0.041 (0.076)	.589	0.105	
Depression (SCL-90-R)									
PRS-ES (*P <* .001)	Intrafamilial adversity	0.161 (0.550)	.770	1.438 (0.836)	.087	0.572 (0.536)	.287	0.090	
	Deprivation	0.440 (0.548)	.423	2.240 (0.857)	.010	−0.369 (0.615)	.550	0.073	
	Threat	0.172 (0.518)	.740	3.537 (0.829)	.000	−0.183 (0.551)	.741	0.188	
PRS-ES (*P <* .01)	Intrafamilial adversity	−0.162 (0.245)	.508	0.802 (1.323)	.545	0.292 (0.255)	.253	0.093	
	Deprivation	−0.079 (0.248)	.752	0.506 (1.498)	.736	0.311 (0.308)	.314	0.074	
	Threat	−0.210 (0.228)	.359	0.648 (1.258)	.607	0.651 (0.269)	.016 (.197)	0.215	DS S
PRS-ES (*P <* .05)	Intrafamilial adversity	−0.038 (0.118)	.749	0.585 (1.009)	.563	0.207 (0.112)	.068	0.100	
	Deprivation	−0.019 (0.119)	.876	1.928 (1.159)	.098	−0.006 (0.139)	.967	0.068	
	Threat	−0.066 (0.112)	.558	1.860 (1.084)	.088	0.206 (0.126)	.104	0.201	
PRS-ES (*P <* .10)	Intrafamilial adversity	0.037 (0.096)	.697	0.782 (1.062)	.462	0.140 (0.095)	.141	0.096	
	Deprivation	0.037 (0.097)	.703	2.071 (1.193)	.084	−0.022 (0.114)	.848	0.069	
	Threat	−0.009 (0.091)	.919	1.697 (1.095)	.123	0.177 (0.102)	.085	0.200	

^a^Adjusted for ancestry PC1 and PC2.

^b^Complete outputs of the LEGIT competitive-confirmatory analyses are shown in Supplementary tables 2–5.

*Note*. PRS-ES, Polygenic Risk Score of Environmental Sensitivity; CAPE, Community Assessment of Psychic Experiences; WSS, Wisconsin Schizotypy Scales; SCL-9-R, Symptom Checklist-90-Revised; Est, Estimate; SE, Standard Error; GxE, Gene-by-environment interaction; DS, Differential Susceptibility; S, Strong; W, Weak.

**Fig. 1.**
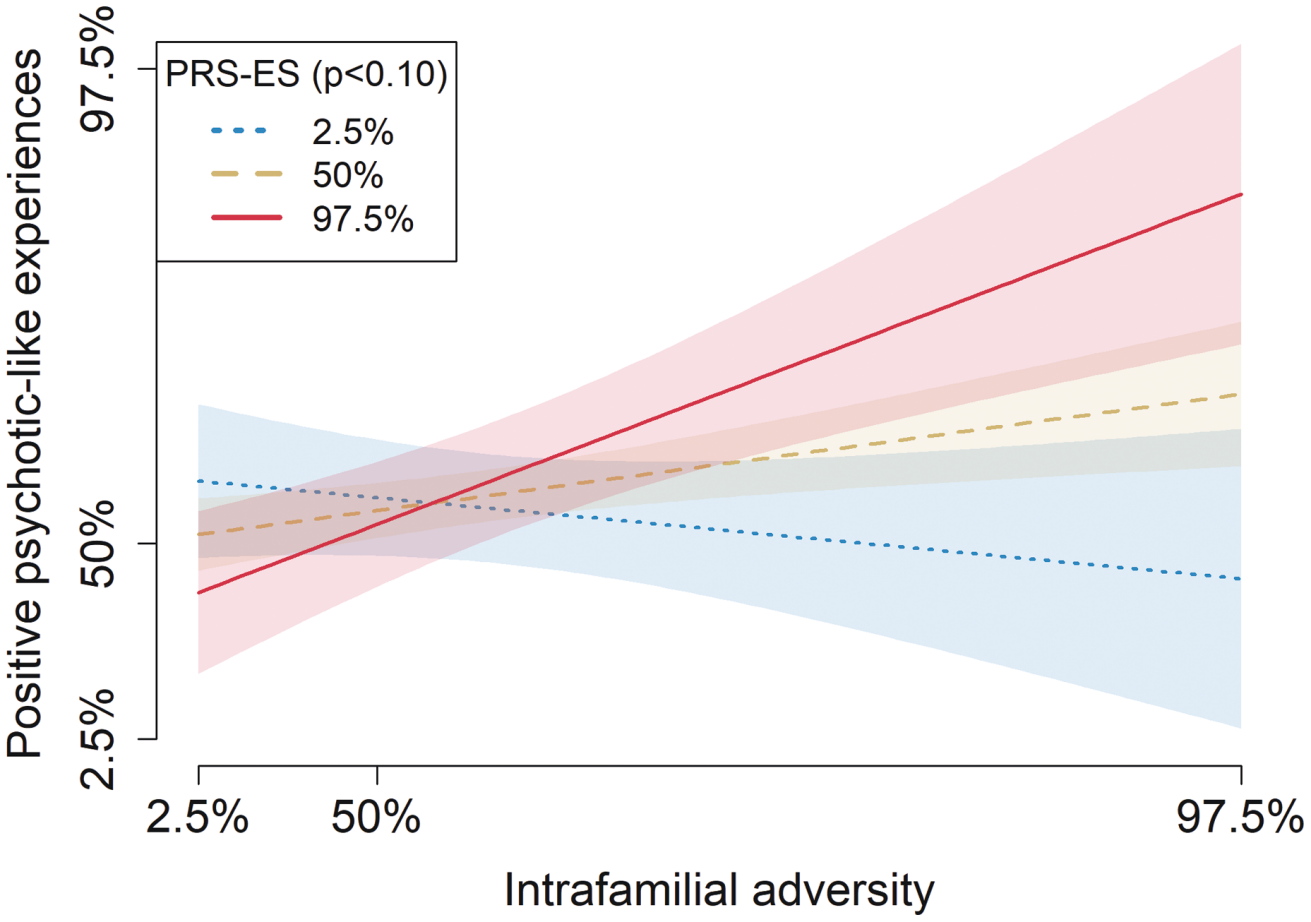
Graphic representation of the best-fitted GxE model for PRS-ES and Intrafamilial Adversity on positive PLE. *Note*: PRS-ES, Polygenic Risk Score Environmental Sensitivity.

Only significant interactions between PRS-ES and Intrafamilial adversity on PLE survived a subsequent FDR correction (please see graphic representation for 1 of the 3 significant thresholds in figure 1).

### PRS-PLE as a Moderating Susceptibility Factor

Regarding exploratory analyses for PRS-PLE, table 2 shows that the interaction of PRS-PLE and Intrafamilial Adversity on PLE (threshold *P* < .10) was consistent with a model of strong DS, and that the interaction of PRS-PLE and Threat on positive schizotypy (threshold *P* < .001) and depression (thresholds *P* < .05; .10) were consistent with a model of weak DS—except for threshold *P* < .05 on depression for which competitive-confirmatory tests could not fit the interaction in any of the GxE models and classified the effect as “Environment only.” PRS-PLE moderated the association between Intrafamilial Adversity and Anxiety (threshold *P* < .05) fitting a diathesis-stress model; however, those with lower PRS-PLE were more affected by the environmental effects. PRS-PLE (threshold *P* < .01) moderated the association between Deprivation and positive psychotic-like experiences, positive schizotypy, and depression, all showing models of both weak DS and diathesis stress; however, those with low PRS-PLE were more affected by Deprivation. None of the interactions with PRS-PLE survived FDR correction.

**Table 2.** Effects of PRS-PLE, Childhood Adversity, and Their Interaction on Subclinical Psychosis Spectrum, Anxiety, and Depression

		PRS		Childhood adversity		PRS × Childhood adversity		*R* ^2^	Best GxE model^b^
		Est. (*SE*)	P	Est. (*SE*)	*P*	Est. (*SE*)^a^	*P* (*P*_FDR_)		
Psychosis spectrum									
Positive Psychotic-like Experiences (CAPE)									
PRS-PLE (*P <* .001)	Intrafamilial adversity	0.934 (0.965)	.335	1.213 (0.381)	.002	0.931 (1.147)	.418	0.058	
	Deprivation	0.329 (0.953)	.730	1.283 (0.356)	.003	−0.170 (1.139)	.882	0.072	
	Threat	0.857 (0.916)	.351	1.906 (0.383)	.000	1.834 (1.047)	.081	0.124	
PRS-PLE (*P <* .01)	Intrafamilial adversity	0.584 (0.487)	.232	0.854 (0.403)	.036	0.613 (0.589)	.291	0.063	
	Deprivation	0.303 (0.475)	.524	1.851 (0.442)	.000	−1.195 (0.564)	.035 (.212)	0.096	DS W
	Threat	0.505 (0.471)	.285	1.624 (0.367)	.000	−0.185 (0.613)	.764	0.114	
PRS-PLE (*P <* .05)	Intrafamilial adversity	0.006 (0.317)	.984	0.599 (0.510)	.241	0.530 (0.391)	.177	0.061	
	Deprivation	−0.057 (0.307)	.853	2.011 (0.509)	.000	−0.639 (0.337)	.060	0.089	
	Threat	0.087 (0.308)	.777	1.570 (0.420)	.000	0.041 (0.410)	.920	0.108	
PRS-PLE (*P <* .10)	Intrafamilial adversity	0.326 (0.284)	.253	0.312 (0.498)	.531	0.734 (0.344)	.034 (.212)	0.077	DS S
	Deprivation	0.138 (0.281)	.624	1.931 (0.595)	.001	−0.522 (0.391)	.184	0.082	
	Threat	0.301 (0.276)	.277	1.411 (0.458)	.002	0.221 (0.373)	.555	0.114	
Positive Schizotypy (WSS)									
PRS-PLE (*P <* .001)	Intrafamilial adversity	0.041 (0.173)	.810	0.204 (0.068)	.003	0.223 (0.205)	.278	0.052	
	Deprivation	−0.080 (0.169)	.637	0.229 (0.063)	.000	−0.060 (0.203)	.769	0.073	
	Threat	0.033 (0.163)	.841	0.334 (0.068)	.000	0.444 (0.187)	.019 (.203)	0.118	DS W
PRS-PLE (*P <* .01)	Intrafamilial adversity	−0.046 (0.087)	.599	0.112 (0.072)	.120	0.189 (0.103)	.068	0.066	
	Deprivation	−0.109 (0.084)	.196	0.335 (0.079)	.000	−0.208 (0.100)	.040 (.203)	0.098	Diathesis-stress W
	Threat	−0.059 (0.085)	.483	0.241 (0.66)	.000	0.066 (0.110)	.550	0.096	
PRS-PLE (*P <* .05)	Intrafamilial adversity	−0.048 (0.056)	.400	0.080 (0.091)	.377	0.107 (0.070)	.124	0.064	
	Deprivation	−0.062 (0.054)	.255	0.359 (0.090)	.000	−0.116 (0.060)	.054	0.095	
	Threat	−0.036 (0.055)	.517	0.244 (0.075)	.001	0.016 (0.073)	.829	0.094	
PRS-PLE (*P <* .10)	Intrafamilial adversity	0.004 (0.051)	.935	0.069 (0.089)	.442	0.102 (0.062)	.099	0.060	
	Deprivation	−0.026 (0.050)	.609	0.348 (0.106)	.001	−0.097 (0.070)	.166	0.082	
	Threat	0.003 (0.050)	.952	0.228 (0.082)	.006	0.033 (0.067)	.625	0.093	
Negative Schizotypy (WSS)									
PRS-PLE (*P <* .001)	Intrafamilial adversity	0.122 (0.210)	.561	−0.036 (0.083)	.663	−0.031 (0.250)	.902	0.025	
	Deprivation	0.008 (0.203)	.969	0.195 (0.076)	.011	−0.412 (0.243)	.091	0.075	
	Threat	0.100 (0.200)	.618	0.239 (0.084)	.005	−0.080 (0.229)	.727	0.082	
PRS-PLE (*P <* .01)	Intrafamilial adversity	−0.018 (0.106)	.865	−0.007 (0.088)	.933	−0.068 (0.126)	.592	0.025	
	Deprivation	−0.060 (0.103)	.562	0.316 (0.096)	.001	−0.208 (0.089)	.089	0.076	
	Threat	−0.027 (0.103)	.795	0.267 (0.080)	.001	−0.052 (0.134)	.698	0.081	
PRS-PLE (*P <* .05)	Intrafamilial adversity	−0.070 (0.069)	.307	0.032 (0.111)	.776	−0.059 (0.085)	.488	0.030	
	Deprivation	−0.077 (0.066)	.243	0.353 (0.109)	.001	−0.124 (0.073)	.088	0.081	
	Threat	−0.058 (0.067)	.387	0.269 (0.091)	.004	−0.029 (0.089)	.743	0.083	
PRS-PLE (*P <* .10)	Intrafamilial adversity	−0.022 (0.062)	.721	0.047 (0.109)	.669	−0.075 (0.076)	.325	0.028	
	Deprivation	−0.035 (0.061)	.566	0.351 (0.128)	.007	−0.111 (0.084)	.188	0.070	
	Threat	−0.013 (0.060)	.834	0.275 (0.100)	.006	−0.026 (0.081)	.751	0.080	
Anxiety (SCL-90-R)									
PRS-PLE (*P <* .001)	Intrafamilial adversity	0.927 (1.103)	.402	1.100 (0.436)	.012	−1.322 (1.311)	.315	0.066	
	Deprivation	0.661 (1.100)	.548	1.162 (0.412)	.005	−1.185 (1.318)	.370	0.057	
	Threat	1.287 (1.051)	.223	2.124 (0.440)	.000	1.987 (1.202)	.100	0.121	
PRS-PLE (*P <* .01)	Intrafamilial adversity	−0.381 (0.560)	.497	1.527 (0.463)	.001	−0.660 (0.665)	.322	0.061	
	Deprivation	−0.428 (0.555)	.441	1.748 (0.516)	.001	−0.995 (0.659)	.133	0.063	
	Threat	−0.183 (0.543)	.737	1.705 (0.424)	.000	0.355 (0.708)	.617	0.105	
PRS-PLE (*P <* .05)	Intrafamilial adversity	−0.863 (0.357)	.017	2.279 (0.574)	.000	−0.965 (0.440)	.030 (.356)	0.097	Diathesis-stress W
	Deprivation	−0.648 (0.354)	.069	1.584 (0.590)	.008	−0.278 (0.391)	.478	0.069	
	Threat	−0.458 (0.351)	.194	1.520 (0.479)	.002	0.399 (0.468)	.395	0.117	
PRS-PLE (*P <* .10)	Intrafamilial adversity	−0.542 (0.327)	.099	2.040 (0.571)	.000	−0.694 (0.394)	.080	0.078	
	Deprivation	−0.418 (0.327)	.202	1.255 (0.691)	.071	0.017 (0.455)	.971	0.059	
	Threat	−0.283 (0.317)	.373	1.380 (0.525)	.009	0.484 (0.260)	.260	0.114	
Depression (SCL-90-R)									
PRS-PLE (*P <* .001)	Intrafamilial adversity	1.895 (1.551)	.223	2.015 (0.614)	.001	−0.722 (1.845)	.696	0.094	
	Deprivation	1.402 (1.559)	.370	1.786 (0.583)	.003	−0.629 (1.866)	.736	0.073	
	Threat	2.027 (1.434)	.159	3.614 (0.601)	.000	1.615 (1.640)	.326	0.198	
PRS-PLE (*P <* .01)	Intrafamilial adversity	0.220 (0.787)	.780	2.452 (0.652)	.000	−0.999 (0.935)	.287	0.091	
	Deprivation	0.126 (0.780)	.872	2.832 (0.726)	.000	−2.022 (0.927)	.030 (.162)	0.092	DS W
	Threat	0.548 (0.737)	.459	3.138 (0.575)	.000	0.852 (0.960)	.376	0.192	
PRS-PLE (*P <* .05)	Intrafamilial adversity	−0.529 (0.512)	.303	2.680 (0.822)	.001	−0.535 (0.397)	.397	0.091	
	Deprivation	−0.343 (0.505)	.497	2.459 (0.841)	.004	−0.509 (0.557)	.362	0.074	
	Threat	0.073 (0.476)	.879	2.554 (0.476)	.000	1.308 (0.634)	.040 (.162)	0.205	E only
PRS-PLE (*P <* .10)	Intrafamilial adversity	0.039 (0.465)	.934	2.329 (0.812)	.005	−0.205 (0.561)	.715	0.085	
	Deprivation	0.091 (0.465)	.845	1.883 (0.982)	.057	−0.009 (0.647)	.989	0.069	
	Threat	0.367 (0.429)	.393	2.295 (0.709)	.001	1.273 (0.578)	.029 (.162)	0.208	DS W

^a^Adjusted for ancestry PC1 and PC2.

^b^Complete outputs of the LEGIT competitive-confirmatory analyses are shown in Supplementary tables 6–9.

*Note*. PRS-PLE, Polygenic Risk Score of Psychotic-like Experiences; CAPE, Community Assessment of Psychic Experiences; WSS, Wisconsin Schizotypy Scales; SCL-9-R, Symptom Checklist-90-Revised; Est, Estimate; SE, Standard Error; GxE, Gene-by-environment interaction; DS, Differential Susceptibility; S, Strong; W, Weak.

## Discussion

To our knowledge, this is the first study examining DS along the psychosis spectrum. Findings partly supported the DS model as individuals with high environmental genetic sensitivity showed increased levels of subclinical psychosis, depression, and anxiety expressions if they experienced high levels of childhood adversity and fewer symptoms if they reported low or no levels of adversity, compared with those with low PRS-ES. Secondarily, the PRS-PLE also moderated associations between childhood adversity and later symptomatology following a DS pattern, most notably for psychosis and, to a lesser extent, depression dimensions but these moderations did not survive FDR.

As hypothesized the majority of significant GxE interactions using PRS-ES were consistent with DS (excepting negative schizotypy), supporting the idea that sensitivity to the environment and psychosocial exposures are relevant causative factors to myriad psychopathological expressions—even if some factors would be more relevant for specific dimensions. Stronger DS effects were found for PLE and positive schizotypy, and results remained significant for PLE and suggestive for positive schizotypy after FDR correction. This result supports the suggested continuum of mental disorders severity,^28^ in which psychotic-like manifestations index greater deviance; thus, greater environmental sensitivity in combination with greater adversity may contribute to stronger effects for such phenotypes. However, here variability related to PRS-ES moderated the impact of adversity on PLE in a “for better and for worse” manner, suggesting that genetics of DS to the environment are relevant to anxiety and depression, as well as psychosis risk expression. This supports the idea that the genetic bases of mental disorders may (partly) reflect genetic variability in environmental sensitivity.

Consistent with the role of adverse psychosocial influences in risk for developing positive symptoms,^50,70^ their severity,^71^ and impact on outcome and course,^72^ we found that DS models predicted positive, but not negative, psychosis dimensions. This finding supports the hypothesis that a heightened affective/stress-sensitivity pathway is relevant to the positive dimension.^73,74^ As hypothesized, we found no significant interactions between PRS-ES and adversity to negative schizotypy. Given that this dimension is characterized by diminished motivation and low openness to experience,^52,75^ we did not expect that a proxy genetic score of environmental sensitivity would moderate variability in negative schizotypy expression.

Research has recently examined whether *positive* psychosocial factors could also impact psychosis risk and outcome. Findings about the protective effect of an absence of adverse childhood experiences in genetically sensitive individuals are consistent with recent epidemiological studies showing the protective role of positive experiences^35^ and studies indicating that positive psychology interventions lower psychosis expression.^76,77^

Regarding the second exploratory goal, we examined DS using PRS-PLE for the first time. Similar to PRS-ES, PRS-PLE moderated the impact of maltreatment on PLE and positive (but not negative) schizotypy, and depression, in a DS pattern. However, the interactions with PRS-PLE did not survive FDR correction. Belsky and Widaman,^66^ however, advocated for eschewing the use of strict *P* values in the exploratory phase and using other less restrictive parameters, which suggests that the subsequent model testing phase may be feasible. The use of the conventional low-powered manner of testing interaction significance may be responsible for the failure to detect more subtle GxE effects in previous research^78^ but could have led to false positives. Given the exploratory nature of the present study along with the limited sample size, a more conservative statistical approach based on the conventional *P* < .05 threshold was employed. Additionally, unlike some GxE studies using a single PRS and environmental predictor,^79–82^ this study examined several models by testing 2 PRS at 4 different evidence-based thresholds and 3 types of adversity, which required applying multiple testing correction procedures based on conventional *P*-values. Although not significant after correction, the effect sizes we found may indicate that genetic variants related to PLE index transdiagnostic risk and resilience for mental suffering as shown with PRS-Schizophrenia^21^ and thus suggest that part of the variance of PRS-PLE may also capture environmental sensitivity.

The pattern of findings with PRS-PLE partially mirror those obtained with PRS-ES, although PRS-PLE yields a more mixed picture. As expected, PRS-ES detected DS effects across several symptom dimensions, whereas PRS-PLE yielded DS effects for PLE and positive schizotypy, with depression showing a weak DS model and no effects for anxiety. This pattern seems consistent with the psychopathology severity continuum hypothesis, in which nonaffective psychosis manifestations index the extreme end of a severity continuum.^28^ Within the psychosis spectrum, strong DS was supported for positive PLE, while weak DS was detected for positive schizotypy. This likely reflects that PLE was the phenotype used to develop the PRS-PLE, which focuses on symptom-like experiences of delusions and hallucinations^49^ rather than milder perceptual abnormalities and magical ideation characterizing schizotypy. Altogether, this picture of findings is consistent with the possibility that PRS-PLE captures both specific disorder-related factors as well as sensitivity to environment.

Regarding the impact of different types of adversity, most interactions were driven by Intrafamilial Adversity and Threat. This is not surprising considering that emotional abuse loaded on both factors as subscales were not forced to load on a single factor^59^—consistent with evidence of substantial co-occurrence of different adversity subtypes,^83,84^ also referred to as polyvictimization.^85^ Intrafamilial Adversity included threatening experiences that primarily pertained to the family domain (eg, parental discord, role reversal, parental violence, and parental antipathy), while the Threat factor also included physical abuse and bullying. In contrast, Deprivation did not yield significant interactions with PRS-ES.

Our findings support claims that genetic liability for psychosis is partially driven by DS to environmental psychosocial insults that affect brain functioning,^86^ and extend them by highlighting the need for integration of positive exposure impact. Homberg and Jagiellowicz^87^ recently pointed out that studying outcomes in both negative and positive environments *simultaneously* may explain some inconclusive findings in GxE research, and advanced models of neural mechanisms involved in DS. Specifically, sensitive individuals exhibit hyperactivity in brain regions involved in the salience network (ie, increased bottom-up processing of exogenous stimuli) and less-efficient inhibition in the central executive network (ie, decreased top-down control over stimuli) which may lead to a more “permissive” neural state to both negative and positive environmental influences.^87^

### Strengths and Limitations

Most previous studies testing DS employed sets of dopaminergic and serotoninergic candidate genes.^88^ In contrast, this study used a PRS-ES indexing plasticity to environment. Recent evidence suggests that PRS show larger cumulative effect sizes and have greater predictive power.^89,90^ Another critical strength of the current study is the combination of self-report with intensive and validated interviews of complementary aspects of childhood experiences. These interviews allowed for contextualized in-depth information that is difficult to tap with self-reports. It contributes to minimizing biases related to subjective responding as ratings rely on objective aspects of experience rather than individual subjective attitudes. However, these high-quality intensive measurements limited our sample size, and thus the ability to detect replicable interaction effects. Nonetheless, the competitive-confirmatory approach used in LEGIT has shown an accuracy of around 70%–85% in similar sample sizes in simulation studies,^64^ compared with 40%–70% with the classic Regions of Significance approach used with similar sample sizes.

Furthermore, the use of a predominantly female university student sample limits generalizability. Thus, replication in community samples with more representative distributions of gender and age would enhance generalizability. Also, absence of adverse childhood experiences was used as a proxy for “positive” environment as employed in previous DS research^91,92^ rather than assessments of specifically positive exposures. Despite this, a notable strength of this sample is that the measurements of PLE and schizotypy dimensions have shown construct,^51^ ecological,^52,93^ and predictive validity over 3^53^ and 10 years.^94^

## Conclusions and Implications

This study showed for the first time that environmental genetic susceptibility moderates the association between childhood adversity and psychosis, affective, and anxiety subclinical experiences consistent with the DS model. That is, participants with high PRS-ES were more reactive to the environment by showing more subclinical symptoms following high levels of adversity but fewer symptoms if not, compared with those with low PRS-ES. Results from the secondary exploratory goal with PRS-PLE, though not surviving statistical correction, depicted a similar pattern. These preliminary findings, if replicated, may support the notion that environmental sensitivity is a key transdiagnostic causative factor of mental suffering. One may speculate that part of this heightened sensitivity could also be captured by specific psychosis-related genetic variants. Although limited statistical power and the exploratory nature of the present study call for replication in larger independent samples, accumulating support for the DS model entails a paradigm shift in schizotypy theory and research. These findings challenge traditional assumptions about vulnerability guided by the diathesis-stress model and call for further consideration of individuals’ environmental susceptibility heterogeneity in etiological research. This should reduce the damaging pessimism surrounding the traditional “heritable broken brain” model in psychopathology, particularly present for psychosis, stressing the potential value of positive exposures, positive psychology interventions, and prevention strategies to decrease the likelihood of poor outcomes in highly sensitive individuals.

## Supplementary Material

Supplementary material is available at https://academic.oup.com/schizophreniabulletin/.

sbad130_suppl_Supplementary_Material
