# Supplementary material for: Genetic Susceptibility to the Environment Moderates the Impact of Childhood Experiences on Psychotic, Depressive, and Anxiety Dimensions
Source: Schizophr Bull. 2025 Mar 4;51(Suppl 2):S95–S106. doi: 10.1093/schbul/sbad130 (PMC11879581; doi:10.1093/schbul/sbad130)
Supplement: sbad130_suppl_Supplementary_Material [file sbad130_suppl_supplementary_material.docx]

**Supplementary Materials**

*Genotyping, Quality Control and Imputation*

DNA was extracted from saliva or cotton swabs using the following extraction kits: i) the prepIT-L2P kit (DNA Genotek Inc., Ottawa, Ontario, Canada) for saliva samples and ii) the RealPure Genomic DNA Extraction Kit (Durviz S.L.U., Valencia, Spain) for cotton swab samples. DNA samples were genotyped at the “Centro Nacional de Genotipado” (CEGEN-PRB3-ISCIII; CNIO-Madrid) using the Illumina Infinium Global Screening Array-24 v2.0 (GSA) BeadChip. Genotype calls were generated with GenomeStudio v2.0.4 (Illumina Inc., San Diego, CA, USA). The quality control (QC) was performed using PLINK v1.9 (www.cog-genomics.org/plink/1.9/)^1^. During QC, SNPs were excluded when: had a missing call rate >2%; had a Minor Allele Frequency (MAF) <0.1%; or deviated from Hardy-Weinberg equilibrium with a P-value <0.001. Subjects were excluded when they had a missing call rate >2%; were genetically related to other participants or duplicated samples according to the pairwise identity by descent method (PI_HAT >0.25); or had non-European ancestry according to a Multidimensional Scaling (MDS) analysis, which was carried out with PLINK v1.9 to obtain a representation of genetic ancestry in our study, extracting the first 10 ancestry components. Seventeen subjects were excluded during QC leaving a sample of 197 subjects. MDS components were recalculated in this final sample and the two first components were used in all models including PRS as independent variables. Imputation was performed using the Haplotype Reference Consortium panel (www.haplotype-reference-consortium.org)^2^ in the Michigan Imputation Server^3^. A post-imputation QC was carried out to exclude SNPs that had an imputation quality score of R2 <0.3; or had a MAF <1%. A total of 7,755,414 SNPs passed post-imputation QC.

**References**

1. Chang CC, Chow CC, Tellier LC, Vattikuti S, Purcell SM, Lee JJ. Second-generation PLINK: rising to the challenge of larger and richer datasets. *Gigascience*. 2015;4:7.
2. McCarthy S, Das S, Kretzschmar W, et al. A reference panel of 64,976 haplotypes for genotype imputation. *Nat Genet*. 2016;48(10):1279-1283.
3. Das S, Forer L, Schönherr S, et al. Next-generation genotype imputation service and methods. *Nat Genet*. 2016;48(10):1284-1287.

**Table 1. Descriptive statistics and Pearson correlations for study variables (N=197).**

|  | *Descriptive statistics* | | *Pearson correlations* | | | | | | | | | | | | | | | |
| --- | --- | --- | --- | --- | --- | --- | --- | --- | --- | --- | --- | --- | --- | --- | --- | --- | --- | --- |
|  | M (SD) | Range | **1** | **2** | **3** | **4** | **5** | **6** | **7** | **8** | **9** | **10** | **11** | **12** | **13** | **14** | **15** | **16** |
| **1.**PRS ES (p<0.001) | 0.91 (1.02) | -2.04 - 3.89 | - | .41*** | .39*** | .37*** | -.050 | -.12 | -.04 | -.05 | .18* | .05 | .10 | -.02 | .08 | .05 | .05 | .08 |
| **2.**PRS ES (p<0.01) | 4.11 (2.27) | -1.55 - 10.76 |  |  | .68*** | .60*** | -.02 | -.04 | -.02 | -.03 | .12 | .08 | .13 | .00 | .05 | -.06 | .02 | -.02 |
| **3.**PRS ES (p<0.05) | 6.83 (4.69) | -5.39 - 20.08 |  |  |  | .89** | -.02 | .01 | -.01 | -.02 | .05 | .08 | .14* | .05 | .11 | .11 | .08 | .01 |
| **4.**PRS ES (p<0.10) | 9.11 (5.73) | -3.31 - 25.15 |  |  |  |  | .03 | .05 | .01 | .00 | .02 | .05 | .12 | .05 | .11 | .11 | .07 | .05 |
| **5.**PRS PLE (p<0.001) | -0.14 (0.36) | -1.18 - 0.78 |  |  |  |  |  | .49*** | .36*** | .35*** | .01 | .13 | .03 | .06 | .01 | .05 | .08 | .10 |
| **6.**PRS PLE (p<0.01) | 0.26 (0.71) | -1.54 - 2.16 |  |  |  |  |  |  | .72*** | .66*** | .07 | .11 | .03 | .09 | -.04 | .01 | -.02 | .06 |
| **7.**PRS PLE (p<0.05) | 0.83 (1.10) | -2.35 - 3.74 |  |  |  |  |  |  |  | .90*** | .11 | .06 | -.04 | .01 | -.05 | -.05 | -.11 | -.02 |
| **8.**PRS PLE (p<0.10) | 1.00 (1.22) | -2.54 - 4.20 |  |  |  |  |  |  |  |  | .07 | .08 | -.01 | .07 | .01 | .01 | -.07 | .04 |
| **9.** Intrafamilial maltreat. | -0.02 (0.97) | -1.15 - 3.01 |  |  |  |  |  |  |  |  |  | .38*** | .32*** | .22** | .20** | -.03 | .23** | .27*** |
| **10.** Deprivation | -0.3 (0.96) | -1.19 - 5.35 |  |  |  |  |  |  |  |  |  |  | .45*** | .26*** | .25*** | .21** | .22** | .24*^**^ |
| **11.** Threat | -0.04 (0.97) | -1.54 – 5.38 |  |  |  |  |  |  |  |  |  |  |  | .32*** | .28*** | .24** | .31*** | .41*** |
| **12.**CAPE Positive PLE | 8.23 (4.82) | 0 - 23 |  |  |  |  |  |  |  |  |  |  |  |  | .75*** | .09 | .52*** | .45*** |
| **13.**WSS Pos schizotypy | -0.34 (0.86) | -1.56 - 2.24 |  |  |  |  |  |  |  |  |  |  |  |  |  | .09 | .54*** | .49** |
| **14.**WSS Neg schizotypy | -0.02 (1.03) | -1.57 - 4.27 |  |  |  |  |  |  |  |  |  |  |  |  |  |  | .13 | .20** |
| **15.**SCL-R-90 Anxiety | 6.67 (5.53) | 0 - 29 |  |  |  |  |  |  |  |  |  |  |  |  |  |  |  | .60** |
| **16.**SCL-R-90 Dep | 11.78 (7.90) | 0 -4 3 |  |  |  |  |  |  |  |  |  |  |  |  |  |  |  |  |

⁎p < 0.05. ⁎⁎ p < 0.01. ⁎⁎⁎ p < 0.001

r>0.30 are medium effect sizes and r>0.50 are large effect sizes.

**Table 2. LEGIT competitive-confirmatory tests for PRS-ES on Positive psychotic-like experiences significant (p<0.05) interactions.**

| *Outcome:*  *Positive psychotic experiences (CAPE)* | | PRS ES (p<.001) x Intrafamilial adversity | | PRS ES (p<.05) x Intrafamilial adversity | | | PRS ES (p<.10) x Intrafamilial adversity | |
| --- | --- | --- | --- | --- | --- | --- | --- | --- |
|  |  | AIC | Crossover point (95%) | AIC | Crossover point (95%) | AIC | | Crossover point (95%) |
| GxE  models | DS STRONG | **1161.92** | **-0.3 (-0.59 / -0.01)** | **1160.77** | **-0.52 (-0.81 / -0.23)** | **1161.38** | | **-0.56 (-0.86 / -0.26)** |
|  | DS WEAK | 1163.82 | -0.29 (-0.57 / 0) | 1162.27 | -0.5 (-0.79 / -0.21) | 1162.8 | | -0.53 (-0.83 / -0.24) |
|  | Diathesis STRONG | 1169.22 | -1 | 1162.39 | -1 | 1162.34 | | -1 |
|  | Diathesis WEAK | 1168.29 | -1 | 1163.99 | -1 | 1164.06 | | -1 |
|  | Vantage STRONG | 1173.04 | 1 | 1174.79 | 1 | 1174.78 | | 1 |
|  | Vantage WEAK | 1166.95 | 1 | 1169.39 | 1 | 1169.46 | | 1 |
| Non-GxE models | Intercept only | 1175.5 | NA | 1175.5 | NA | 1175.5 | | NA |
|  | G only | 1177.46 | NA | 1176.92 | NA | 1177.02 | | NA |
|  | E only | 1167.55 | NA | 1167.55 | NA | 1167.55 | | NA |
|  | G+E only | 1168.92 | NA | 1169.17 | NA | 1169.14 | | NA |

*Note: Best model indicated by lowest AIC is* ***highlighted****.*

**Table 3. LEGIT competitive-confirmatory tests for PRS-ES on Positive schizotypy significant (p<0.05) interactions.**

| *Outcome:*  *Positive schizotypy (WSS)* | | PRS ES (p<.01) x Threat | |
| --- | --- | --- | --- |
|  |  | AIC | Crossover point (95%) |
| GxE  models | DS STRONG | **481.74** | **-0.58 (-0.82 / -0.35)** |
|  | DS WEAK | 483.21 | -0.58 (-0.82 / -0.35) |
|  | Diathesis STRONG | 487.05 | -1 |
|  | Diathesis WEAK | 486.5 | -1 |
|  | Vantage STRONG | 502.59 | 1 |
|  | Vantage WEAK | 488.65 | 1 |
| Non-GxE models | Intercept only | 501.73 | NA |
|  | G only | 503.28 | NA |
|  | E only | 486.71 | NA |
|  | G+E only | 488.68 | NA |

*Note: Best model indicated by lowest AIC is* ***highlighted****.*

**Table 4. LEGIT competitive-confirmatory tests for PRS-ES on Anxiety significant (p<0.05) interactions.**

| *Outcome:*  *Anxiety (SCL-R-90)* | | PRS ES (p<.05) x Intrafamilial adversity | | PRS ES (p<.10) x Intrafamilial adversity | |
| --- | --- | --- | --- | --- | --- |
|  |  | AIC | Crossover point (95%) | AIC | Crossover point (95%) |
| GxE  models | DS STRONG | **1214.71** | **-0.61 (-0.91 / -0.31)** | 1218.15 | -0.66 (-0.99 / -0.32) |
|  | DS WEAK | 1216.48 | -0.58 (-0.88 / -0.29) | 1220.15 | -0.66 (-0.99 / -0.33) |
|  | Diathesis STRONG | 1214.98 | -1 | **1217.64** | -1 |
|  | Diathesis WEAK | 1216.66 | -1 | 1218.98 | -1 |
|  | Vantage STRONG | 1229.98 | 1 | 1230.16 | 1 |
|  | Vantage WEAK | 1223.08 | 1 | 1223.01 | 1 |
| Non-GxE models | Intercept only | 1229.55 | NA | 1229.55 | NA |
|  | G only | 1230.17 | NA | 1230.54 | NA |
|  | E only | 1221.09 | NA | 1221.09 | NA |
|  | G+E only | 1222.02 | NA | 1222.16 | NA |

*Note: Best model indicated by lowest AIC is* ***highlighted****.*

**Table 5. LEGIT competitive-confirmatory tests for PRS-ES on Depression significant (p<0.05) interactions.**

| *Outcome:*  *Depression (SCL-R-90)* | | PRS ES (p<.01) x Threat | |
| --- | --- | --- | --- |
|  |  | AIC | Crossover point (95%) |
| GxE  models | DS STRONG | **1328.95** | **-0.47 (-0.64 / -0.3)** |
|  | DS WEAK | 1330.83 | -0.46 (-0.63 / -0.29) |
|  | Diathesis STRONG | 1349.37 | -1 |
|  | Diathesis WEAK | 1336.96 | -1 |
|  | Vantage STRONG | 1364.88 | 1 |
|  | Vantage WEAK | 1334.68 | 1 |
| Non-GxE models | Intercept only | 1369.4 | NA |
|  | G only | 1371.32 | NA |
|  | E only | 1335.03 | NA |
|  | G+E only | 1335.72 | NA |

*Note: Best model indicated by lowest AIC is* ***highlighted****.*

**Table 6. LEGIT competitive-confirmatory tests for PRS-PLE on Positive psychotic-like experiences significant (p<0.05) interactions.**

| *Outcome:*  *Positive psychotic experiences (CAPE)* | | PRS PLE (p<.01) x Deprivation | | PRS PLE (p<.10) x Intrafamilial adversity | |
| --- | --- | --- | --- | --- | --- |
|  |  | AIC | Crossover point (95%) | AIC | Crossover point (95%) |
| GxE  models | DS STRONG | 1177.54 | -1.27 (-3.42 / 0.88) | **1164.61** | **-0.65 (-0.98 / -0.32)** |
|  | DS WEAK | **1162.1** | **-0.56 (-0.88 / -0.23)** | 1166.2 | -0.67 (-1 / -0.35) |
|  | Diathesis STRONG | 1175.67 | -1 | 1165.71 | -1 |
|  | Diathesis WEAK | 1164.98 | -1 | 1165.56 | -1 |
|  | Vantage STRONG | 1176.29 | 1 | 1177.39 | 1 |
|  | Vantage WEAK | 1163.91 | 1 | 1169.49 | 1 |
| Non-GxE models | Intercept only | 1175.5 | NA | 1175.5 | NA |
|  | G only | 1175.94 | NA | 1176.5 | NA |
|  | E only | 1163.3 | NA | 1167.55 | NA |
|  | G+E only | 1164.54 | NA | 1168.9 | NA |

*Note: Best model indicated by lowest AIC is* ***highlighted****.*

**Table 7. LEGIT competitive-confirmatory tests for PRS-PLE on Positive schizotypy significant (p<0.05) interactions.**

| *Outcome:*  *Positive schizotypy (WSS)* | | PRS PLE (p<.01) x Deprivation | | PRS PLE (p<.001) x Threat | |
| --- | --- | --- | --- | --- | --- |
|  |  | AIC | Crossover point (95%) | AIC | Crossover point (95%) |
| GxE  models | DS STRONG | 504.71 | -0.39 (-2.12 / 1.35) | 505.72 | -1.68 (-75.48 / 72.12) |
|  | DS WEAK | 488.6 | -0.8 (-1.12 / -0.48) | **485.18** | **-0.58 (-0.83 / -0.33)** |
|  | Diathesis STRONG | 503.72 | -1 | 503.72 | -1 |
|  | Diathesis WEAK | **487.55** | **-1** | 487.35 | -1 |
|  | Vantage STRONG | 503.14 | 1 | 503.72 | 1 |
|  | Vantage WEAK | 491.32 | 1 | 488.53 | 1 |
| Non-GxE models | Intercept only | 501.73 | NA | 501.73 | NA |
|  | G only | 503.34 | NA | 503.72 | NA |
|  | E only | 489.9 | NA | 486.71 | NA |
|  | G+E only | 490.74 | NA | 488.7 | NA |

*Note: Best model indicated by lowest AIC is* ***highlighted****.*

**Table 8. LEGIT competitive-confirmatory tests for PRS-PLE on Anxiety significant (p<0.05) interactions.**

| *Outcome:*  *Anxiety (SCL-R-90)* | | PRS PLE (p<.05) x Intrafamilial adversity | |
| --- | --- | --- | --- |
|  |  | AIC | Crossover point (95%) |
| GxE  models | DS STRONG | 1230.06 | 0.35 (-0.84 / 1.54) |
|  | DS WEAK | 1216.62 | -0.88 (-1.19 / -0.56) |
|  | Diathesis STRONG | 1231.46 | -1 |
|  | Diathesis WEAK | **1214.82** | **-1** |
|  | Vantage STRONG | 1228.25 | 1 |
|  | Vantage WEAK | 1221.19 | 1 |
| Non-GxE models | Intercept only | 1229.55 | NA |
|  | G only | 1229.14 | NA |
|  | E only | 1221.09 | NA |
|  | G+E only | 1219.26 | NA |

*Note: Best model indicated by lowest AIC is* ***highlighted****.*

**Table 9. LEGIT competitive-confirmatory tests for PRS-PLE on Depression significant (p<0.05) interactions.**

| *Outcome:*  *Depression (SCL-R-90)* | | PRS PLE (p<.01) x Deprivation | | PRS PLE (p<.05) x Threat | | PRS PLE (p<.10) x Threat | |  |
| --- | --- | --- | --- | --- | --- | --- | --- | --- |
|  |  | AIC | Crossover point (95%) | AIC | Crossover point (95%) | AIC | Crossover point (95%) |  |
| GxE  models | DS STRONG | 1372.53 | -1.27 (-4.5 / 1.95) | 1349.32 | -0.59 (-0.83 / -0.34) | 1343.24 | -0.63 (-0.84 / -0.41) | |
|  | DS WEAK | **1359.13** | **-0.61 (-0.96 / -0.25)** | 1335.56 | -0.59 (-0.77 / -0.4) | **1334.38** | **-0.67 (-0.85 / -0.48)** | |
|  | Diathesis STRONG | 1370.58 | -1 | 1365.7 | -1 | 1358.59 | -1 | |
|  | Diathesis WEAK | 1361.05 | -1 | 1336.4 | -1 | 1334.56 | -1 | |
|  | Vantage STRONG | 1370.86 | 1 | 1370.21 | 1 | 1371.3 | 1 | |
|  | Vantage WEAK | 1361.22 | 1 | 1336.92 | 1 | 1336.83 | 1 | |
| Non-GxE models | Intercept only | 1369.4 | NA | 1369.4 | NA | 1369.4 | NA | |
|  | G only | 1370.71 | NA | 1371.3 | NA | 1371.02 | NA | |
|  | E only | 1359.84 | NA | **1335.03** | **NA** | 1335.03 | NA | |
|  | G+E only | 1361.61 | NA | 1337.02 | NA | 1336.51 | NA | |

*Note: Best model indicated by lowest AIC is* ***highlighted****.*
